# Evaluation of Radiographic Pattern of Male Urethral Strictures in Nigeria: A Preliminary Report of a Proposed New Scoring System for Developing Countries

**DOI:** 10.5812/iranjradiol.6357

**Published:** 2012-03-25

**Authors:** Ahmed Ahidjo, Adekunle Yishau Abdulkadir, Ibrahim Ahmed Gadams, Suleiman Tanimu Saad

**Affiliations:** 1 Department of Radiology, University of Maiduguri Teaching Hospital, Maiduguri, Nigeria; 2 Department of Radiology, Federal Medical Center Gusau, Gusau, Nigeria; 3 Department of Surgery, University of Maiduguri Teaching Hospital, Maiduguri, Nigeria; 4 Department of Radiology, Federal Medical Center Gombe, Gombe, Nigeria

**Keywords:** Urethral Stricture, Male, Nigeria, Developing Countries

Dear Editor,

Urethral stricture is a common urological problem affecting the male gender [1][2][3][4][5]. There is currently progressive increase in the incidence of this condition due to the improved living standards, the increased number of permanent catheter bearers, the surge of sexually transmitted diseases’ (STDs) incidence, and misuse of transurethral diagnostic or therapeutic instrumentation [1][2][3][4][5]. Studies have shown that the peak prevalence is at 40-45years of age and is very rare below 9 years [2][3][4][5][6]. In developing countries, infection is the predominant cause of stricture unlike the developed world where trauma is predominate [2][3][4][5][6].

Radiology is the basis of confirmation of strictures. Conventional imaging of the urethra with a dynamic Retrograde Urethro-Cystography (RUCG) is an easy to perform, readily available, reproducible and cost-effective examination that can detect clinically relevant strictures involving the anterior urethra and those with extension into the membranous urethra [7][8][9][10][11] making it still the initial imaging of choice for suspected stricture disease in the developing countries. Currently, there is no standardized format for reporting urethrograms by a radiologist. The aim of this study was to determine the radiographic pattern of male urethral stricture and to propose a new scoring system to assist the radiologist by introducing a standard format for reporting urethral strictures on RUCG and to help the urologist in decision making for the management of patients with urethral strictures.

A multicenter retrospective review of the urethrograms and medical records of all patients who had a RUCG at the University of Maiduguri Teaching Hospital and Gombe Federal Medical Center, Nigeria from January 2006 to December 2009 was carried out.

**Figure 1:**
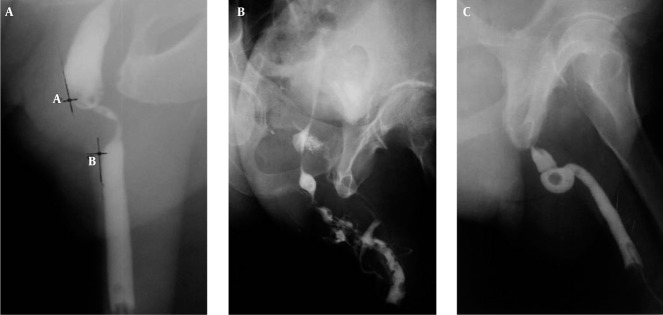
A, A 40-year-old man presented with difficulty in passing urine. Urethrogram performed demonstrates a peno-bulbar stricture and how measurement of the stricture length was done. A-B is the stricture length; B, A 62-year-old man presented with difficulty in passing urine and multiple sessions of bougienation in the past. Urethrogram revealed multiple strictures with varying degrees of narrowing with a fistula and prostatic extravasation; C, A 48-year-old man presented with acute urinary retention to the emergency department. There was a positive history of progressive difficulty in passing urine. Urethrogram revealed complete urethral stricture showing an abrupt termination of contrast and bulbar dilatation. The rounded lucency is a radiolucent calculus.

Radiographic patterns of strictures which included the number, site, types, length and associated radiographic findings as well as complications were obtained from the contrast urethrograms. All patients who had an RUCG done using the technique described by Kawashima et al. [9] were included in the study. The stricture length was measured as the maximum distance along a tangential straight line touching the edges of the normal urethra adjoining the stricture segment either above or below as shown in Figure 1A.

The study is divided into two phases. Phase 1 included evaluation of the radiographic findings in the male urethral stricture and formulation of the scoring system. Phase 2 of the research was mainly to assess clinical usefulness and accuracy of the scoring system.

The data collected for phase 1 of the research was entered into a computer and analyzed using SPSS 11.0 for Windows (SPSS Inc., Chicago Illinois).

A total of 103 urethrocystograms satisfied our inclusion criteria. The age of patients ranged from 20 years to 78 years with the mean of 43.40±18.50 years. About 64.8% of the strictures occurred between the ages of 30 and 59 years with peak age incidence of 42 years. The strictures involved the anterior urethra in 99 patients (96.1%), the majority of which involved the bulbar region. They were multiple (Figure 1B) in 68.9% of cases, while single in 31.1%. The observed length of strictures ranged between 2mm and 25mm in 74.3%. Infection was the cause in 74%. The observed pathologic associations with strictures included calculus (Figure 1C), intravasation of contrast, diverticulum, vesico-ureteric reflux and fistula.

According to McCallum [10], it is no longer sufficient for the radiologist to delineate a tight stricture in the urethra and leave the operative decision solely to the urologist. Rather the radiologist should direct the attention of the urologist to the appropriate management required. Therefore, we proposed a Gombe Urethrographic Scoring of urethral strictures based on the radiographic findings on urethrography relevant to the urologist. The major aim of this scoring system is to assist the radiologist in reporting urethrographic findings relevant to the urologist in deciding treatment options. This may also assist the urologist in correlating the urethrographic scores with treatment decisions and outcomes.

The details of the scoring system are mentioned below:

I. Normalcy; 1, normal urethrogram; 2, abnormal urethrogram

II. Number: 1, one stricture; 2, two strictures; 3, more than two strictures.

III. Location: 1, anterior location; 2, posterior location; 3, involving both anterior and posterior.

IV. Length: 1, short segment stricture; 2, medium segment stricture; 3, long segment stricture.

V. Narrowing: 1, minimal stricture; 2, tight stricture; 3,complete or total occlusion.

VI. Complication: 1, uncomplicated; 2, single complication; 3, multiple complications.

Strictures less than 1 cm are considered short, between 1 and 4cm are medium and more than 4cm are long segment strictures. The least score is 1 and the highest score is 17. A score of 1 reveals a normal urethrogram and 2-17 is an abnormal urethrogram which may require surgical intervention. In grading our scoring system, we take into consideration the available treatment options of urethral strictures;

A, dilatation or urethrotomy

B, resection and anastomosis

C, augmentation or replacement

Based on our proposed scoring system, patients with scores of 2-5 may benefit from treatment option A, 6-9 will benefit from treatment option B and scores of 10-17 may benefit from treatment option C; however, replacement is advisable for complete urethral strictures. This scoring of urethrographic findings may be of value to the radiologists in recording all the relevant radiologic features during film reporting and probably the urologists in management decision. We wish to state categorically that this proposal needs to be tested for clinical use and we have already started the second phase of the research to ascertain the clinical relevance of the proposed scoring system.
